# Application of CEEMD noise reduction algorithm in ultrasound imaging in evaluating fetuses with abnormal glucose metabolism in late pregnancy

**DOI:** 10.12669/pjms.37.6-WIT.4844

**Published:** 2021

**Authors:** Junfeng Huang, Cuiting Wang, Xianxia Li, Yuqin Jing

**Affiliations:** 1 Junfeng Huang, Deputy Chief Nurse. Department of Nursing, Maternal and Child Health Hospital of Jinan City, Jinan City 250001, Shandong Province, China; 2 Cuiting Wang, Bachelor’s Degrees. Department of Obstetrical, Maternal and Child Health Hospital of Jinan City, Jinan City 250001, Shandong Province, China; 3 Xianxia Li, Supervisor nurse. Department of Obstetrical, Maternal and Child Health Hospital of Jinan City, Jinan City 250001, Shandong Province, China; 4 Yuqin Jing, Supervisor nurse. Department of Surgical, Maternal and Child Health Hospital of Jinan City, Jinan City 250001, Shandong Province, China

**Keywords:** Glucose metabolism, Late pregnancy, Receiver operating characteristic, Measured fetal brain artery, Umbilical artery

## Abstract

**Objectives:**

To explore the predictive effect of abnormal glucose metabolism and fetal hemodynamic parameters on adverse pregnancy outcome.

**Methods:**

One hundred and nine pregnant women with abnormal glucose metabolism during pregnancy from June 2016 to October 2018 were selected and divided into poor prognosis group (34 cases) and good prognosis group (75 cases). The hemodynamic parameters of fetal cerebral artery (MCA), umbilical artery (UA) and uterine artery of pregnancy (UT-A), including peak systolic velocity (s / D), resistance index (RI) and plasticity index (PI), were measured by color Doppler ultrasound. The receiver operating characteristic (ROC) curve of adverse pregnancy outcomes was drawn and the best threshold index was determined.

**Results:**

MCA-PI poor prognosis group, MCA-RI, RI ratio (MCA/UA) are lower than the good prognosis group, Ut-A-PI is higher than the good prognosis group (P<0.05,). ROC curve analysis results show that when the MCA-PI 1.56, the sensitivity of the predicted adverse outcomes of pregnancy, the highest specificity<, was 91.18%, 80.00%, respectively. Logistic regression analysis of risk factors shows poor pregnancy outcomes include: pregnant women, older age, body mass index ≥24.0kg/m2 and a family history of diabetes. Protective factors include exercise during pregnancy, MCA-PI≥1.56, MCA-RI≥0.63 and RI The ratio (MCA/UA) ≥0.84.

**Conclusion:**

Color Doppler ultrasound measured MCA-PI<1.56 the most important indicators of poor pregnancy outcomes as abnormal glucose metabolism during pregnancy and predict the exact cutoff. Pregnant women, older age, body mass index ≥24.0kg/m2 and a family history of diabetes and abnormal glucose metabolism during pregnancy risk factors for adverse outcomes of pregnancy.

## INTRODUCTION

Abnormal glucose metabolism during pregnancy includes diabetes with pregnancy and gestational diabetes. Its incidence has increased in recent years, which is a common complication during pregnancy, resulting in 4 times the perinatal morbidity and mortality compared to normal pregnancy. Abnormal glucose metabolism during pregnancy can reduce the placenta’s blood supply to the fetus, and the fetus is in a state of high blood sugar and high insulin, which will cause the body to consume more oxygen, cause fetal hypoxia, and restrict growth,. and fetal health. Because ultrasound can dynamically monitor the fetal blood circulation in the uterus, the study of fetal hemodynamics by ultrasound has become very popular in recent years.^1^-^3^ This study used color Doppler ultrasound to measure the hemodynamic parameters of pregnant women with abnormal glucose metabolism during pregnancy and their fetuses, to determine the most important indicators and cutoffs for predicting adverse pregnancy outcomes. Our objective was to study factors affecting adverse pregnancy outcomes to prevent their occurrence.

## METHODS

A total of 109 pregnant women with abnormal glucose metabolism during pregnancy who were treated in our hospital from June 2016 to October 2018 were selected. There were 34 cases with good prognosis. The pregnant women were 20-42 years old, with an average of (27.68±3.93) years old. Mean gestational age was 38 check +6 week’s gestation. Delivery methods are supposed to ensure smooth process and safe delivery.

Any one or more of the following is considered to be a poor pregnancy outcome: 1. newborn body weight> 90th percentile of the same gestational week; 2. primary cesarean section; 3. clinical neonatal hypoglycemia; 4. neonatal hyperinsulinemia; 5. premature delivery; 6. neonatal hyperbilirubinemia; 7. fetal distress; 8. neonatal asphyxia; 9. amniotic fluid turbidity above II degree or oligohydramnios.^4^,^5^

Philips iU 22 using color Doppler ultrasound, C5-2 abdominal probe, the frequency of 3.0-5.0 MHz Checking pregnant holding supine position, fully exposed to the abdomen, were measured by color Doppler ultrasound fetal brain artery (the MCA), hemodynamic parameters umbilical artery (UA) and pregnant uterine artery (Ut-A), comprising: late peak systolic velocity/systolic blood flow velocity (S/D), the resistance index (RI) and pulsatility index (PI). 1. UA: Use two-dimensional ultrasound to locate the free umbilical cord, and choose the sampling point as the filling point of blood flow signal. 2. MCA: Use two-dimensional ultrasound to find the fetal head. When the fetal biparietal standard measurement section is displayed, move the probe parallel to the skull base direction. Use color Doppler ultrasound to clearly display the fetal middle cerebral artery when sampling. point. 3. Ut-A: Look up the internal iliac artery from the groin on both sides of the pregnant woman and find the main uterine artery from the distal branch of the internal iliac artery. During the measurement, adjust the color Doppler sampling volume to 2mm, keep the angle between the pulse sampling line and the blood vessel at 0-30°, freeze the image when more than five continuous stable standard waveforms are obtained, and take the average of three consecutive measurements. The signal sampling time does not exceed one minute.

## RESULTS

Comparison of hemodynamic parameters of pregnant women and fetuses between two groups Poor prognosis group MCA-PI, MCA-RI and RI ratio (MCA/UA) are lower than the good prognosis group, Ut-A-PI is higher than the good prognosis group, the differences were statistically significant (P<0.05); the remaining each parameter difference was not statistically significant. Table-I.^6^

**Table-I:** Comparison of hemodynamic parameters of pregnant women and fetuses between two groups (*x*±*s*).

*Group*	*MCA*			*UA*			*Ut-A*		

	*PI*	*RI*	*S/D*	*PI*	*RI*	*S/D*	*PI*	*RI*	*S/D*
Poor	1.51±0.12	0.53±0.10	3.30±0.77	1.21±0.25	0.74±0.12	3.67±0.94	0.84±0.12	0.61±0.08	2.28±0.20
Good	1.61±0.11	0.63±0.09	3.34±0.48	1.17±0.13	0.71±0.12	3.51±1.11	0.76±0.13	0.58±0.07	2.24±0.21
t	3.788	4.958	0.661	0.981	1.18	1.387	3.192	1.498	0.868
P	0	0	0.509	0.329	0.241	0.166	0.002	0.137	0.868
*Group*
	*PI ratio*	*RI ratio*	*S/D ratio*	*PI ratio*	*RI ratio*	*S/D ratio*	*PI ratio*	*RI ratio*	*S/D ratio*

	*(MCA/UA)*	*(MCA/UA)*	*(MCA/UA)*	*(MCA/UA)*	*(MCA/UA)*	*(MCA/UA)*	*(MCA/UA)*	*(MCA/UA)*	*(MCA/UA)*
Poor	1.30±0.27	0.72±0.13	0.94±0.26	0.84±0.30	1.02±0.26	0.77±0.22	0.68±0.12	0.75±0.12	0.84±0.24
Good	1.39±0.18	0.90±0.15	1.01±0.25	0.91±0.23	0.98±0.27	0.76±0.28	0.67±0.14	0.74±0.12	0.85±0.27
t	1.582	6.418	0.108	0.121	1.312	1.221	1.449	1.453	1.691
P	0.117	0	0.198	0.167	0.15	0.159	0.142	0.156	0.103

MCA: middle cerebral artery; UA: umbilical artery; Ut-A: uterine artery; PI: plasticity index; RI: resistance index; S/D: peak systolic flow rate/end-systolic blood flow velocity.

###  Analysis of ROC curve

**Fig.1:**
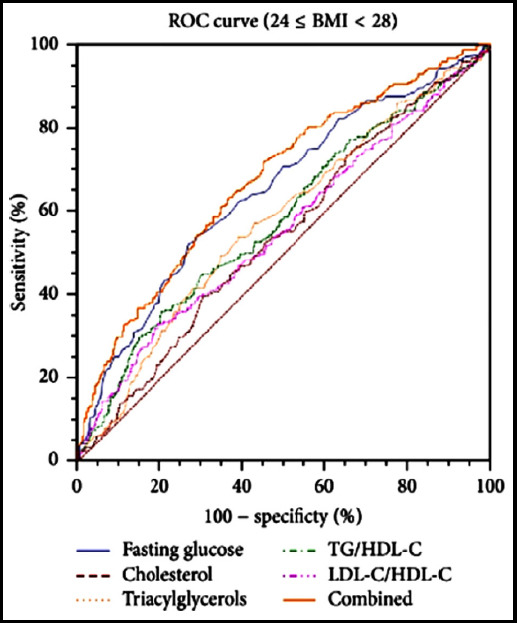
ROC curve of various parameters predicting adverse pregnancy outcomes due to abnormal glucose metabolism during pregnancy.

With MCA-PI<1.56 as the cut-off value, the sensitivity and specificity of predicting adverse pregnancy outcomes of abnormal glucose metabolism during pregnancy are high, 91.18% and 80.00%, respectively, and the area under the curve is 0.800, Table-II and Fig.1.

**Table-II:** Predictive effects of various hemodynamic parameters on adverse pregnancy outcomes during abnormal glucose metabolism during pregnancy.

*Hemodynamic parameters*	*Area under the curve*	*95%*	*P*	*Critical*	*Sensitivity (%)*	*Specificity (%)*	*Yoden index*
MCA-PI	0.8	0.702~0.899	0	<1.56	91.18	80	0.712
MCA-RI	0.767	0.668~0.865	0	<0.63	47.06	96	0.431
Ut-A-PI	0.704	0.590~0.817	0.001	≥0.72	44.12	85.33	0.295
RI ratio (MCA/UA)	0.832	0.749~0.915	0	<0.84	67.65	92	0.596

MCA: cerebral arteries; Ut-A: uterine arteries; UA: umbilical arteries; PI: pulse index; RI: resistance index.

### Univariate analysis

Related characteristics were compared, results showed that: two groups were statistically significant (all P<0.05) in age, high-density lipoprotein, body mass index, family history of diabetes during pregnancy and whether the movement, the difference. Table-III.

**Table-III:** Comparison of two groups of general information.

*Group*	*Family history of diabetes (example)*		*Family history of hypertension (example)*		*Exercise during pregnancy (example)*		*Body mass index (kg/m2)*	

	*no*	*Have*	*no*	*Have*	*no*	*Have*	*≤23.9*	*≥24.0*
Poor prognosis group (75)	49	26	52	23	52	23	34	41
Good prognosis group (34)	32	2	23	11	11	23	27	7
χ^2^/t/Z	10.154		0.031		13.116		11.024	
P	0.001		0.86		0.001		0.001	

## DISCUSSION

In this study, the hemodynamic parameters of fetal middle cerebral artery of some pregnant women with abnormal glucose metabolism during pregnancy changed compared with those of normal pregnant women. The blood flow resistance index PI, RI, S/D was lower than that of normal pregnant women, but no adverse outcome occurred.^7^-^10^

Results of this study MCA-PI poor prognosis group, MCARI, RI ratio (MCA/UA) are lower than the good prognosis group, Ut-A-PI poor prognosis group than good prognosis group, the differences were statistically significant (all P<0.05), in line with cerebral protection effect, consistent with the findings. When MCA-PI<1.56 is taken as the critical value, the sensitivity and specificity were 91.18%, 80.00%, area under the curve 0.800, consistent with previous studies^11^-^13^.

Studies by Jugovic et al. Showed that the decrease in RI ratio (MCA/UA) was related to intrauterine hypoxia. Fetal babies with RI ratio (MCA/UA)<1 were five minutes after birth, Apgar score, umbilical artery blood gas acid-base status, and oxygen. Fetal partial pressures are significantly lower than those of fetuses with RI ratio (MCA/UA)>1, which was similar to the results of this work^14^-^15^. In addition, studies proved that the umbilical artery has a certain application value in the evaluation of fetal hypoxia. When fetal hypoxia occurs, the blood flow of the umbilical artery decreases significantly and the blood flow resistance index increases significantly^16^-^18^. The results of this study are consistent with those of the above investigators.

Studies have suggested that pregnant women with a family history of diabetes have a five times higher risk of abnormal pregnancy outcomes than pregnant women without a family history of diabetes^19^. In addition, prolonged maternal hyperglycemia causes abnormal hyperplasia of fetal islet B cells and adipocytes, leading to obesity and abnormal glucose metabolism in neonates, which result in adverse pregnancy outcomes.^20^

Exercise during pregnancy, MCA-PI ≥ 1.56, MCA-RI ≥ 0.63, and RI ratio (MCA/UA) ≥ 0.84 are protective factors for pregnancy prognosis. At present, the index of S/D of umbilical artery is still widely used in clinic, and the S/D>3 of fetal umbilical blood flow in late pregnancy indicates the possibility of fetal intrauterine hypoxia, but the results are not satisfactory. Therefore, it is still an important task to improve the accuracy of predicting the adverse pregnancy outcomes of abnormal glucose metabolism during pregnancy. Accurate prediction of adverse pregnancy outcomes due to abnormal glucose metabolism during pregnancy can not only reduce adverse pregnancy outcomes such as neonatal encephalopathy, neonatal asphyxia and neonatal death, but also reduce unnecessary cesarean deliveries due to clinical prediction errors. In this study, ROC curve analysis of different hemodynamic parameters and adverse pregnancy outcomes showed that in a single hemodynamic parameter, MCA-PI, MCA-RI, and UT-A-RI can all be used as indicators to predict adverse pregnancy outcomes. However, among the combined parameters, only RI ratio (UA/MCA) could be used as a predictor of adverse pregnancy outcomes. The ROC curve analysis in this study showed that the accuracy of combined hemodynamic parameter RI ratio (UA/MCA) was better than that of single hemodynamic parameter MCA-PI. When MCA-PI ≥ 1.56, MCA-RI ≥ 0.63, and RI ratio (MCA/UA) ≥ 0.84, the fetal blood circulation in the mother’s body conforms to the physiological state of the fetal stage. Currently, MCA hemodynamics is in a high-impedance state, and peripheral blood vessels UA and Ut-A are in a low-resistance state. When they are abnormal, the fetus appears hypoxic, which causes poor pregnancy outcomes such as amniotic fluid pollution.

### Limitations of the study

Due to the time, morbidity, and the fact that the hospital is not a specialist obstetrics and gynecology hospital, the sample size is limited and the number of cases in the pregestational diabetes group is small, which results in a certain degree of deviation in the research results. Therefore, the researchers will continue to collect as many cases as possible in the future work for further in-depth study.

## CONCLUSIONS

Color Doppler ultrasound can be used to obtain hemodynamic parameters to predict adverse pregnancy outcomes in pregnant women with abnormal glucose metabolism during pregnancy, assess fetal intrauterine conditions, and provide clinical ultrasound to predict adverse pregnancy outcomes in patients with abnormal glucose metabolism during pregnancy. Quantitative indicators; comprehensive evaluation of the influencing factors of abnormal glucose metabolism during pregnancy will help early detection of intrauterine abnormalities in the fetus and prevent adverse pregnancy outcomes.

### Authors’ Contribution:

**JH:** Conceived the study, literature review, data analysis, drafting the manuscript.

**CW & XL:** Helped in design, data collection, drafting the manuscript & critical revision.

**YJ:** takes the responsibility and is accountable for all aspects of the work in ensuring that questions related to the accuracy or integrity of any part of the work are appropriately investigated and resolved.
